# Effect of HSP90AB1 and CC domain interaction on Bcr-Abl protein cytoplasm localization and function in chronic myeloid leukemia cells

**DOI:** 10.1186/s12964-021-00752-9

**Published:** 2021-07-03

**Authors:** Yuhang Peng, Zhenglan Huang, Fangzhu Zhou, Teng Wang, Ke Mou, Wenli Feng

**Affiliations:** Department of Clinical Hematology, Key Laboratory of Laboratory Medical Diagnostics Designated By Ministry of Education, School of Laboratory Medicine, Chongqing Medical University, No.1, Yixueyuan Road, Yuzhong District, Chongqing, 400016 China

**Keywords:** Chronic myeloid leukemia, Nuclear localization, Bcr-Abl, HSP90AB1, Coiled-coil domain

## Abstract

**Background:**

The fusion oncoprotein Bcr-Abl is mostly located in the cytoplasm, which causes chronic myeloid leukemia (CML). After moving into the nucleus, the fusion protein can induce apoptosis of CML cells. The coiled-coil domain (CC domain) of Bcr-Abl protein plays a central role in the subcellular localization. However, how CC domain affects subcellular localization of Bcr-Abl remains unclear.

**Methods:**

Herein, the key proteins interacting with the Bcr-Abl CC domain were screened by immunoprecipitation binding mass spectrometry. The specific site of Bcr-Abl CC domain binding to target protein was predicted by Deep Viewer. Immunoprecipitation assay was used to confirmed the specific sites of protein binding. IF and western blot were used to observe the subcellular localization of target protein. Western blot was used to examine the protein changes. CCK-8, clonal formation test and FCM cycle detection were used to observe the effect of inhibitor on the proliferation ability of CML cells. FCM apoptosis detection was used to observe the level of cells apoptosis.

**Results:**

HSP90AB1 interacts with Bcr-Abl CC domain via N-terminal domain (NTD), preventing the transport of Bcr-Abl protein to the nucleus and maintaining the activation of Bcr-Abl tyrosine kinase. The nucleus-entrapped Bcr-Abl markedly inhibits the proliferation and induces apoptosis of CML cells by activating p73 and repressing the expression of cytoplasmic oncogenic signaling pathways mediated by Bcr-Abl. Moreover, the combination of 17AAG (Tanespimycin) with Leptomycin B (LMB) considerably decreased the proliferation of CML cells.

**Conclusion:**

Our study provides evidence that it is feasible to transport Bcr-Abl into the nucleus as an alternative strategy for the treatment of CML, and targeting the NTD of HSP90AB1 to inhibit the interaction with Bcr-Abl is more accurate for the development and application of HSP90 inhibitor in the treatment of CML and other Bcr-Abl-addicted malignancies.

**Video abstract**

**Supplementary Information:**

The online version contains supplementary material available at 10.1186/s12964-021-00752-9.

## Background

Chronic myeloid leukemia (CML) is a myeloid leukemia subtype represented by the formation of Bcr-Abl fusion gene [1, 2]. This fusion gene plays a critical role in CML pathology, encoding Bcr-Abl oncoprotein which accommodates tyrosine kinase activity that can activate multiple downstream signal targets involved in the regulation of cell malignant proliferation and apoptosis, including JAK–STAT [3, 4], RAS-MAPK [5] and CRKL [6, 7]. Imatinib, the first-line tyrosine kinase inhibitor (TKI) (IM), dasatinib and nilotinib, the second-generation kinase inhibitors are remarkably effective treatments for patients in chronic phase [8–11]. However, the occurrence of drug resistance or disease relapse urgently need the development of alternative treatments [12, 13].

Bcr-Abl oncoprotein primarily localized in the cytoplasm. It has the same effector domain as c-Abl whereas c-Abl shuttles between the cytoplasm and nucleus [14]. Based on our previous work, we have designed a drug transduction system to direct the oncogenic Bcr-Abl into the nucleus and induce the apoptosis of CML cells by tyrosine kinase activity [15]. Similar studies also have shown that Bcr-Abl induces the apoptosis of CML cells when transported into the nucleus [16–18]. Notably, the localization of Bcr-Abl plays a crucial role in the development of CML disease, and the coiled-coil domain (CC domain) at the N-terminal of Bcr-Abl is a major determinant for the location in the cytoplasm [19, 20]. We had constructed the pAdTrack-Bcr/Abl-ΔCC expression vector which successfully induces Bcr-Abl transportation into the nucleus after its transfection into 293 T cells (Additional file 1: Figure S1). However, how CC domain affects subcellular localization of Bcr-Abl remains unclear.

As one of the molecular chaperones, Heat Shock Protein 90 (HSP90) functions to facilitate the correct folding of synthesized and denatured oncogenic proteins that participate in leukemia, including Bcr-Abl and its downstream signaling partners [21–23]. Therefore, the dependence on HSP90 has promoted the anti-leukemia drug development by depleting the molecular chaperone and degrading oncogenic Bcr-Abl, therefore eliciting apoptosis of leukemia cells [24–26]. Previous studies have shown that inhibition of the N- and C-terminal termini can disrupt HSP90 chaperone function and cause the degradation of Bcr-Abl oncoprotein. Both 17-AAG, the N-terminal inhibitor and cisplatin, the C-terminal inhibitor have the capacity to suppress progenitor cells and deplete the leukemia stem cells [27–29]. However, the role of HSP90, which affects the subcellular localization of Bcr-Abl, has not been reported.

HSP90AB1, also recognized as HSP90 beta, is a member of the HSP90 family which includes HSP90 alpha (HSP90AA1) and HSP90 beta. HSP90 proteins play an important role in cell regulation, forming complexes with various transcription factors, cellular kinases, and some molecules [30–32]. In this study, we have discovered the effect of the interaction between HSP90AB1 and the CC domain on Bcr-Abl cytoplasm localization and its function in chronic myeloid leukemia cells. After confirming the binding of CC domain with HSP90AB1, we explored the specific binding sites of HSP90AB1 with Bcr-Abl. Further studies have suggested that Bcr-Abl enters the nucleus after destruction of the HSP90AB1-Bcr/Abl complex. The activation of downstream signaling molecule of Bcr-Abl was downregulated. Similarly, the nucleus-entrapped Bcr-Abl causes the activation of p73 and its downstream signaling molecules, markedly inducing the apoptosis and inhibiting the proliferation of CML cells. In our study, we explored the interaction between CC domain and HSP90AB1, and Bcr-Abl was translocated into the nucleus after dissociation with HSP90AB1. Moreover, we elucidated the mechanism of apoptosis induced by the HSP90 inhibitor after Bcr-Abl was translocated into the nucleus. Our study also found the effect of targeted killing of CML cells is enhanced under the action of 17AAG and LMB inhibitors.

## Materials and methods

### Cell lines and cell culture

K562 (Cell Bank of Shanghai Institute of Cell Biology, Chinese Academy of Sciences) and K562/G01 cell lines were grown and maintained in RPMI-1640 medium supplemented with 10% fetal bovine serum (Gibco, USA). K562/G01 is an imatinib-resistant cell line obtained from K562 treated for several months with persistently increased concentration of imatinib up to 5 mg/L. 293 T cells were grown and maintained in Dulbecco’s modified Eagle medium (DMEM) containing 10% fetal bovine serum. All of these cells were maintained in a humidified atmosphere with 5% CO_2_ at 37 °C.

### Identification of CC domain-interacting partners

293 T cells stably expressing HA-tagged Bcr-Abl were lysed for 30 min and subjected to centrifugation at 12,000 g for 30 min. The collected supernatant was incubated overnight at 4℃ with Protein A/G magnetic beads adsorbed with anti-HA epitopes antibody (Cell Signaling Technology). Immunoprecipitation proteins adsorbed on magnetic beads were eluted for western blot assay, and the location of CC-domain specific interacting proteins on SDS-PAGE gel was found by silver staining. The target strip was cut and the specific proteins were identified by the Protein Facility at the Center of Biomedical Analysis.

### Co-immunoprecipitation (Co-IP) and immunoblot analysis

Protein A/G magnetic beads were washed three times with TBST and then incubated with HA-tagged or DYKDDDDK-tagged antibody (Cell Signaling Technology) at room temperature for 1 h. Protein lysates extracted from 293 T cells were added to Protein A/G magnetic beads and incubated overnight at 4℃. The immunoprecipitation proteins adsorbed on the magnetic beads were eluted for western blot assay, and the interaction between HSP90AB1 protein and the CC domain was observed by western blot.

### Western blotting

Western blotting assay was performed as previously described [15]. We purchased the following antibodies from Cell Signaling Technology of USA: c-ABL(#2862), Phospho-c/ABL(#2864), HA-Tag(#3724), DYKDDDDK-Tag(#8146), PARP(#9532), Caspase-3(#9662), BID(#2002), Phospho-AKT(#9271), AKT(#9272), Phospho-STAT5(#9351), p73(#14620), Bax(#5023) and p21(#2947S). The HSP90-beta antibody (Genetex, #101448 USA) and β-actin antibody (BOSTER, bs-0061R China) were the same as the antibodies used above with 1:1000 dilution.

### Transfection

The plasmid of pAdTrack-Bcr/Abl and pAdTrack-Bcr/Abl-ΔCC was maintained in our laboratory. The pAdTrack-HSP90AB1 and pAdTrack-HSP90AB1-ΔNTD expression plasmid purchased from Addgene. Cells were transfected with the plasmid of HSP90AB1, and BCR-ABL with Lipofactamine2000 (Invitrogen, Carlsbad, CA, USA), according to the protocol. All constructs were verified through DNA sequencing and western blot analysis.

### Immunofluorescence assay

Cells were collected for immunofluorescence assay, washed 3 times by PBS and then coated on slides, fixed (30 min, 4% paraformaldehyde solution), permeabilized (20 min, 0.1% Triton X-100), blocked (2 h, goat serum) and then incubated with primary antibodies (1:500 in goat serum). After incubation with a fluorescent-labeled secondary antibody (Introvigen, USA) in darkness for 1 h at 37 °C, cells were stained with diluted DAPI (15 min, 1:1000 in PBS).

### CCK-8 assay

CML cells in the logarithmic growth phase were taken and added to the 96-well culture plate at a density of 4000 cells per well with 100 μl RPMI 1640 containing 15% fetal bovine serum and cultured at 37 °C in a 5% CO2 humidified incubator. Each well was added with 10 μl CCK-8 (MCE, USA) at the indicated time and incubated for 1 h at 37 °C in darkness. The absorbance at 450 nm was measured on a microplate. Each group included four counterparts and was performed three times.

### Cell Colony-forming assay

K562 and K562/G01 cells were taken and added to the 24-well culture plate at a density of 500 cells per well mixed with RPMI 1640 medium containing 20% serum to form a cell suspension of 750L; 750L 1.8% methylcellulose (Sigma, USA) was added in and thoroughly mixed, with 3 parallel holes in each group. The colony number was observed and counted after being cultured at 37℃ for 10d in a 5% CO2 incubator. The colony formation assay was performed five times.

### Apoptotic and cell cycle analysis

K562 and K562/G01 cells, after being treated with 17AAG, LMB and IM at the indicated concentration, were collected and added into 6-well culture plate at a density of 1 × 10^6^ cells per well. Apoptosis was assessed using an Apoptosis Detection Kit according to the instruction. Moreover, nuclear morphology was examined by DAPI staining, and the results were observed with the fluorescence microscope. The cell cycle was analyzed by PI staining, and quantified by using FCM. The percentage of cells in different phases of the cell cycle was determined and quantitated.

### Statistical methods

All statistical results were shown as mean ± SD. The statistical significance among each group was assessed by one-way ANOVA analysis. Statistical analyses were calculated with GraphPad Prism 5.0 software. Results with statistical significance were marked with asterisks. Statistical significance is indicated as *p* < 0.05.

## Results

### Identification of HSP90AB1 as a Bcr/Abl binding partner

**Fig. 1:**
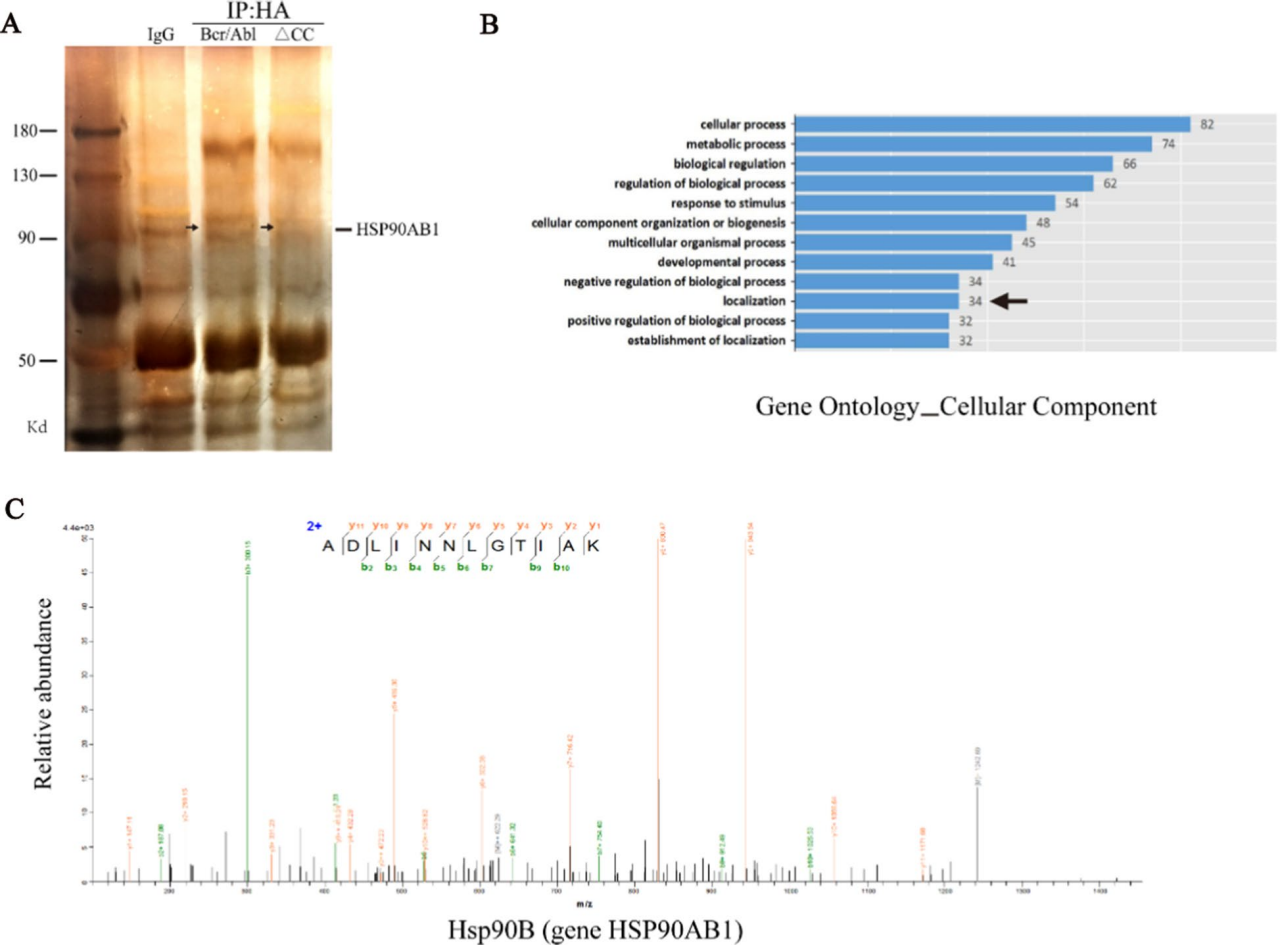
Identification of HSP90AB1 as a Bcr/Abl binding partner. **a** Total cell extracts prepared from 293 T cells expressing HA-tagged Bcr/Abl, HA-tagged Bcr/Abl-ΔCC or vector alone were subjected to immunoprecipitation using anti-HA beads. Proteins were resolved by SDS-PAGE and visualized by silver staining. **b** The proteins selected by LC/MS–MS were classified according to their related functions, and the differential proteins related to protein subcellular localization were screened out through GO_CC. **c** LC/MS–MS spectrometry of the purified HA-Bcr/Abl–associated peptides corresponding to HSP90AB1

In eukaryotic cells, the interaction between proteins affects the subcellular localization of proteins. We hypothesized that certain proteins in the cytoplasm that bind to the CC domain for anchoring the Bcr-Abl protein in the cytoplasm. To identify the potential partners of Bcr-Abl, firstly, we used 293 T cells expressing HA-tagged Bcr/Abl and HA-tagged Bcr/Abl-ΔCC. The location of CC-domain specific interacting proteins on SDS-PAGE gel was found by silver staining (Fig. 1a). The target strip was cut, and the specific proteins were identified by the protein facility, which led to the identification of HSP90AB1 as a differential protein related to protein subcellular localization and associated with the CC domain of Bcr-Abl (Fig. 1b, c; Additional file 2: Table S1).

### Association between the Bcr/Abl CC-domain and N-terminal domain of HSP90AB1

**Fig. 2:**
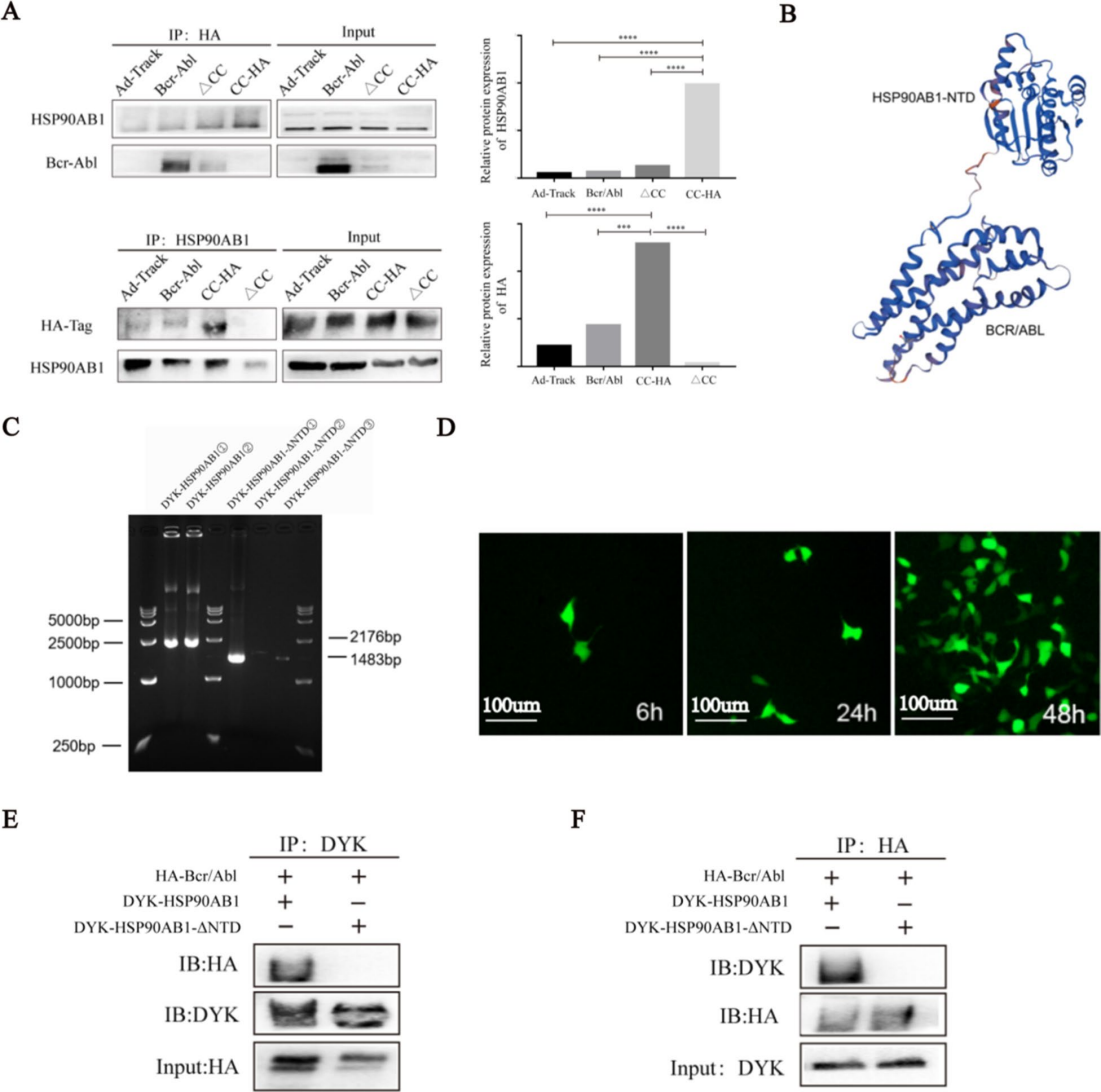
Association between the Bcr/Abl CC-domain and N-terminal domain of HSP90AB1. **a** DYK-tagged HSP90AB1 and/or HA-tagged Bcr-Abl were overexpressed in 293 T cells and subjected to reciprocal coimmunoprecipitation (Co-IP) to detect protein interaction between Bcr-Abl and HSP90AB1. *p < 0.05 and ***p < 0.001. **b** The three-dimensional structure diagram of Bcr/Abl-HSP90AB1 binding protein was simulated using the bioinformatics analysis software Deep Viewer. **c** The target gene expression vectors DYK-pAdTrack-HSP90AB1 and DYK-pAdTrack-HSP90AB1-ΔNTD were successfully constructed by using molecular cloning technology. The target genes HSP90AB1 and HSP90AB1-ΔNTD were amplified by PCR technology, and their bases were 2176 bp and 1483 bp. **d** Immunofluorescence was used to observe the transfection efficiency of the DYK-pAdTrack-HSP90AB1 vector with the fluorescent label in 293 T cells at 6 h, 24 h, and 48 h. **e, f** 293 T cells expressing HA-tagged Bcr-Abl, DYK tagged HSP90AB1 or DYK-HSP90AB1-ΔN were subjected to coimmunoprecipitation using anti-Flag beads

To confirm the intracellular binding of HSP90AB1 and CC domain, we enforced the expression of HA-Bcr/Abl and DYK-HSP90AB1 in 293 T cells for reciprocal immunoprecipitation and confirmed the associations between HSP90AB1 and Bcr-Abl proteins (Fig. 2a). To further explore the specific binding sites of HSP90AB1-Bcr/Abl interaction, we have simulated the three-dimensional structure diagram of the binding protein Bcr/Abl-HSP90AB1, in which the N-terminal region of HSP90AB1 (amino acids 1–693) was predicted to bind to Bcr-Abl (Fig. 2b). To assess the importance of the N-terminal region in mediating Bcr/Abl-HSP90AB1 interaction, we synthesized the DYK-tagged HSP90AB1-ΔNTD expression plasmid (amino acids 694-2176 of HSP90AB1) and DYK-tagged HSP90AB1 expression plasmid to test their reciprocal interaction with Bcr-Abl (Additional file 1: Table S2; Fig. 2c). The transfection efficiency of the target vector in 293 T cells was observed by immunofluorescence, as we transfected DYK-pAdTrack-HSP90AB1 and DYK-pAdTrack-HSP90AB1-ΔNTD with HA-pAdTrack-Bcr/Abl into 293 T cells (Fig. 2d). As expected, the DYK-HSP90AB1 but not the DYK-HSP90AB1-ΔNTD efficiently pulled down Bcr-Abl in HA-tagged Bcr-Abl group, meanwhile, the HA-tagged Bcr-Abl efficiently pulled down DYK-HSP90AB1 (Fig. 2e, f). In a nutshell, those results provide evidence supporting that HSP90AB1 is a direct Bcr/Abl-binding partner, and the N-terminal region of HSP90AB1 (amino acids 1–693) is responsible for the interaction between Bcr-Abl and HSP90AB1.

### Inhibition of HSP90AB1 induces nuclear localization of Bcr/Abl in CML cells

**Fig. 3:**
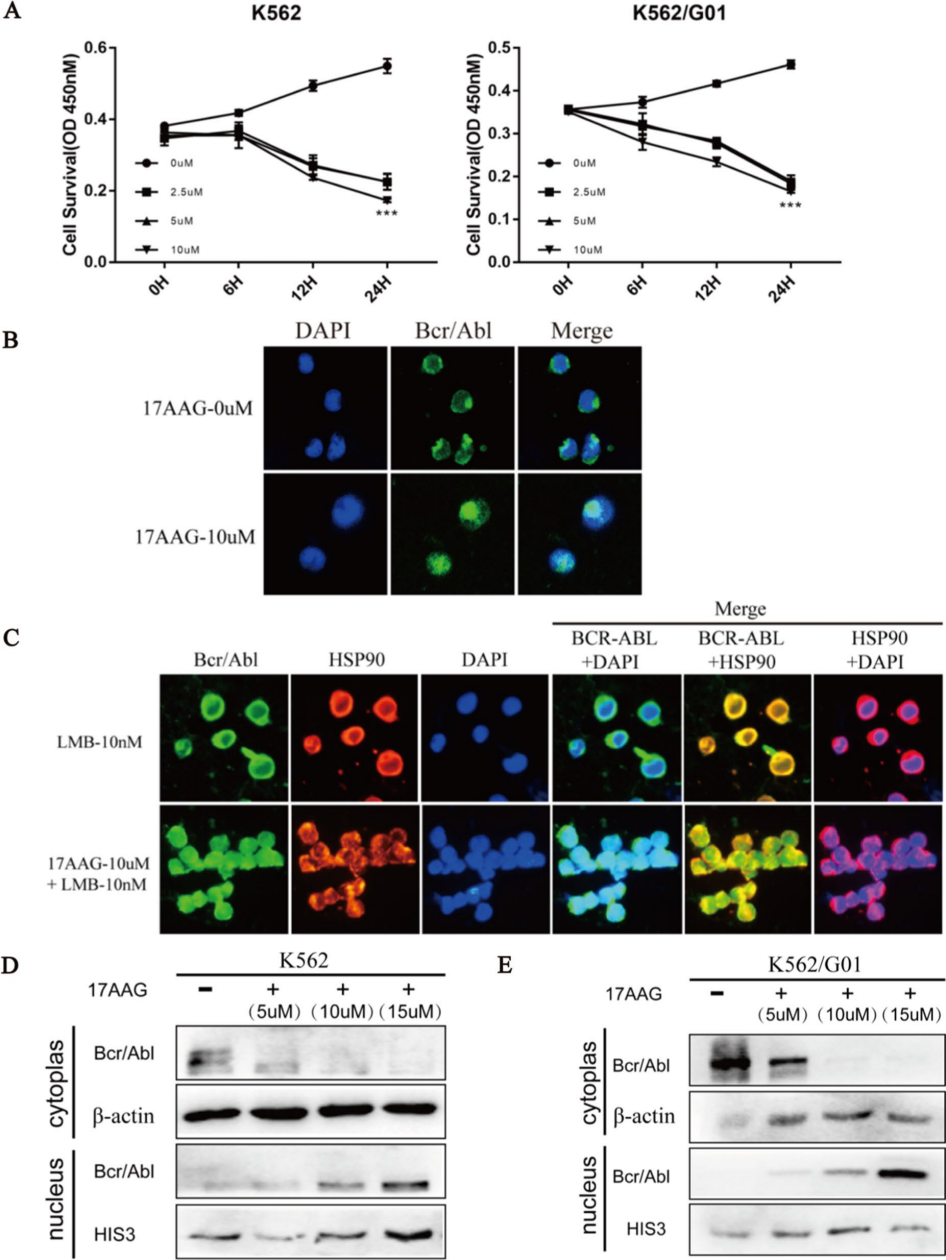
Inhibition of HSP90AB1 induces nuclear localization of Bcr-Abl in CML cells. **a** The concentration of HSP90 inhibitor 17AAG on CML cells was screened by Cell Counting Kit-8 experiment. *p < 0.05, **p < 0.01 and ***p < 0.001. **b** The changes of Bcr-Abl localization in K562 cells treated with HSP90 inhibitor 17AAG were observed by indirect immunofluorescence. **c** The changes of Bcr-Abl localization in K562 cells treated with HSP90 inhibitor 17AAG and protein nuclear export inhibitor LMB were observed by indirect immunofluorescence. **d, e** The changes of Bcr-Abl protein expression in cytoplasm and nucleus of K562 and K562/G01 cells treated with HSP90 inhibitor 17AAG were observed by western blot analyses

17AAG (Tanespimycin) is a classic HSP90 specific N-terminal inhibitors that inhibits the chaperone function of HSP90 and dissociates it from the chaperone protein. The concentration of 17AAG was controlled within 10uM and treated within 6H, which could maintain the CML cells in a relatively stable state and inhibit the function of HSP90AB1 (Fig. 3a). Here, we hypothesized that HSP90AB1 may regulate the localization of Bcr-Abl. To test this hypothesis, we have observed the changes of Bcr-Abl in its subcellular localization after dissociation with HSP90AB1, and after the use of the appropriate concentration of 17AAG (Fig. 3b).

The Bcr-Abl protein has three complete nuclear localization signals and a nuclear output signal, so it can shuttle between the cytoplasm and nucleus [33]. Considering the part of Bcr-Abl was transported out of the nucleus after entering the nucleus, we added protein nucleus output inhibitor LMB (Leptomycin) and HSP90 inhibitor 17AAG to treat K562 cells and observed the changes of Bcr-Abl in its subcellular localization. As shown in Fig. 3c, Bcr-Abl was scattered in the cytoplasm without nucleation in the control group, and there was a co-localization relationship between HSP90AB1 and Bcr-Abl as a molecular chaperone in the cytoplasm. Bcr-Abl showed significant nuclear localization after treatment with 17AAG. This increase in nuclear Bcr-Abl was enhanced by the treatment with 17AAG and LMB. Next, we investigated the subcellular localization of Bcr-Abl by extracting proteins from the cytoplasm and the nucleus and by performing western blot analyses. The expression of Bcr-Abl in the nuclear increased in concentration dependence, with the increasing concentration of HSP90 inhibitor (Fig. 3d, e). The inhibition of HSP90 chaperone function had a significant effect on the subcellular localization of Bcr-Abl, and the formation of HSP90AB1-Bcr/Abl complex also led to the localization of Bcr-Abl protein in the cytoplasm.

### Nuclear transport of Bcr/Abl induced apoptosis in K562 and K562/G01 cells through Bcr/Abl kinase-dependent and Bcr/Abl-independent pathways

**Fig. 4:**
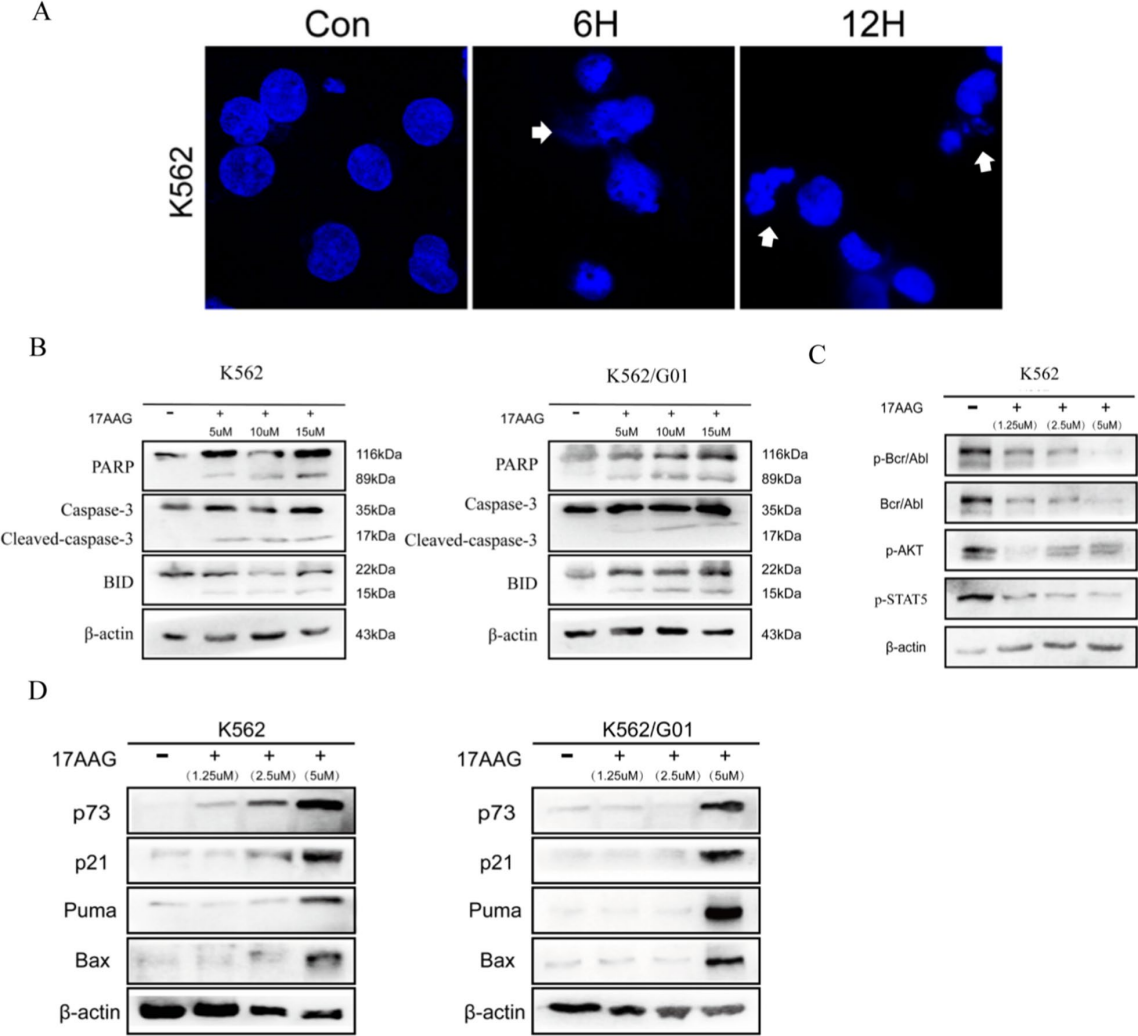
Nuclear transport of Bcr-Abl induced apoptosis in K562 and K562/G01 cells through Bcr-Abl kinase-dependent and Bcr/Abl-independent pathways. **a** The nuclear morphological structure changes of K562 cells were observed by DAPI fluorescence staining after being treated with 17AAG (10 μM) for 6 h, and 12 h. **b** To observe the effect of Bcr-Abl on the activation of apoptosis-related proteins in K562 and K562/G01 cells by western blot analyses. **c** The effect of HSP90 inhibitor 17AAG on the activation of Bcr-Abl, p-Bcr/Abl and downstream signaling molecules such as p-AKT and p-STAT5 in K562 cells. **d** The effect of HSP90 inhibitor 17AAG on the activation of p73 and downstream signaling molecules such as p21, Puma and Bax in K562 and K562/G01 cells

To examine the effect of Bcr-Abl on CML cells after nucleation, we used immunofluorescence assay to observe the changes of the nuclear morphological structure of K562 cells treated with HSP90 inhibitor 17AAG for 6 h and 12 h (Fig. 4a). Some of the nucleus deformed after treatment for 6 h, and some cells showed nuclear fragmentation after treatment for 12 h, suggesting the occur of CML cell apoptosis. Then, we investigated the activation of apoptosis-related proteins in K562 and K562/G01 cells after treatment with 17AAG by using western blot analyses. BID, a bcl-2 family protein, is generated by the hydrolysis of caspase-8 in the Fas signaling pathway and can play a pro-apoptotic role. As predicted, the expression of BID protein increased in a concentration dependent manner in K562 and K562/G01 cells. Cleaved poly ADP-ribose polymerase (PARP) and caspase-3 are also cut into segments during apoptosis, which is also a typical feature of apoptosis (Fig. 4b). It was suggested that Bcr-Abl play a role in promoting apoptosis after entering the nucleus. Next, we observed the changes in activation of Bcr-Abl and its downstream signaling molecules in K562 cells after Bcr-Abl was induced into the nucleus (Fig. 4c). The expression of Bcr-Abl, p-Bcr-Abl and downstream signaling molecules p-AKT, p-STAT5 in K562 cells decreased in a concentration-dependent manner with the increase of inhibitor concentration, which suggested that the expression of downstream signaling molecules of Bcr-Abl was down-regulated, and the malignant proliferation of CML cells was inhibited. Our previous research has reported that nuclear localization of c-Abl causes activation of p73 and induces cell apoptosis [15, 18, 34]. To confirm that Bcr-Abl located in the nucleus plays the same effects on p73 in CML cells, we examined the expression of p73 and its downstream targets. As expected, we found that the level of p73 protein was increased by 17AAG treatment, and the expression of p21, Puma and Bax was also significantly enhanced (Fig. 4d). These results demonstrated that Bcr-Abl located in the nucleus can inhibit the proliferation and induce the apoptosis of CML cells through activating p73 and down-regulating the expression of Bcr-Abl downstream oncogenic proteins.

### Combination of 17AAG and LMB significantly enhances the anti-leukemia effect in vitro

**Fig. 5:**
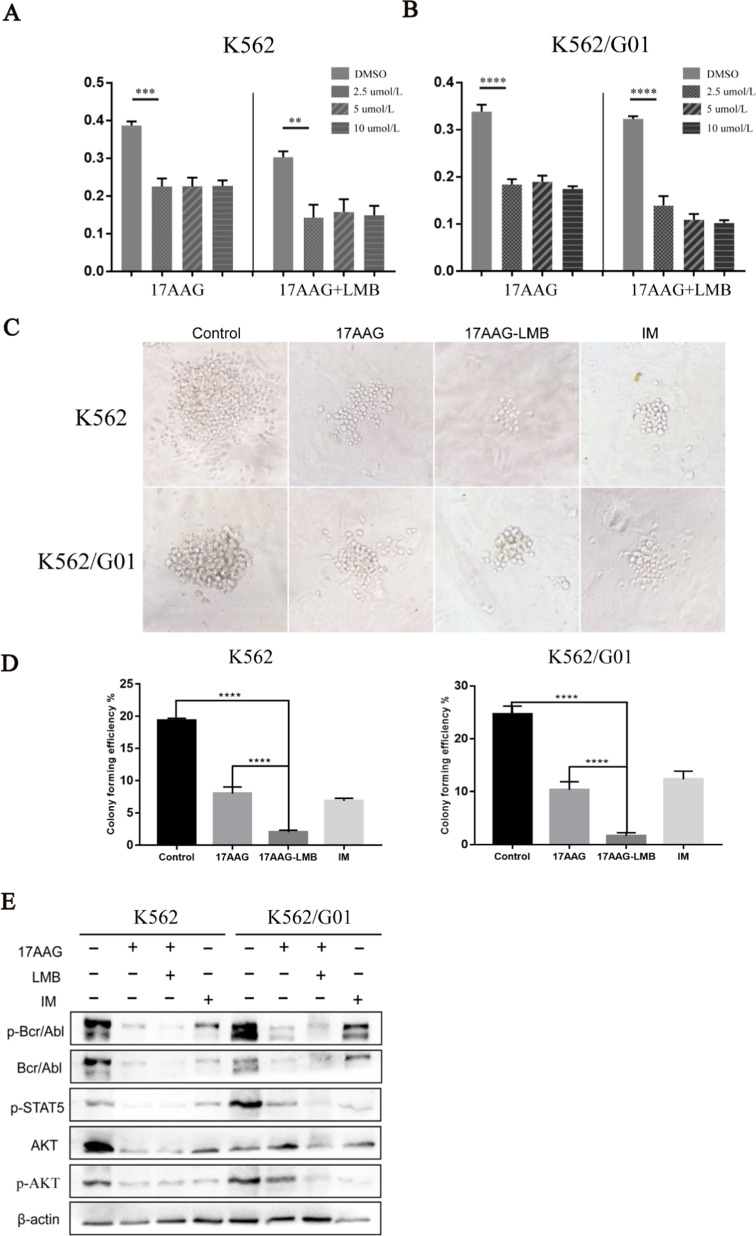
Combinational therapy of 17AAG and LMB significantly inhibits the malignant proliferation of CML cells. **a, b** CCK-8 assay compared the effects of 17AAG with the effects of 17AAG combined with LMB on the proliferation of K562 and K562/G01 cells. **c, d **To observe the effect of drug combination on cell proliferation ability in clone formation experiments and count the number of colonies after being treated with 10 μM 17AAG combined with 10 nM LMB for 5 days. **e** Effects of activation of Bcr-Abl and downstream signaling molecules in K562 and K562/G01 cells were associated with the use of 10 μM 17AAG combined with 10 nM LMB. *p < 0.05, **p < 0.01 and ***p < 0.001

**Fig. 6:**
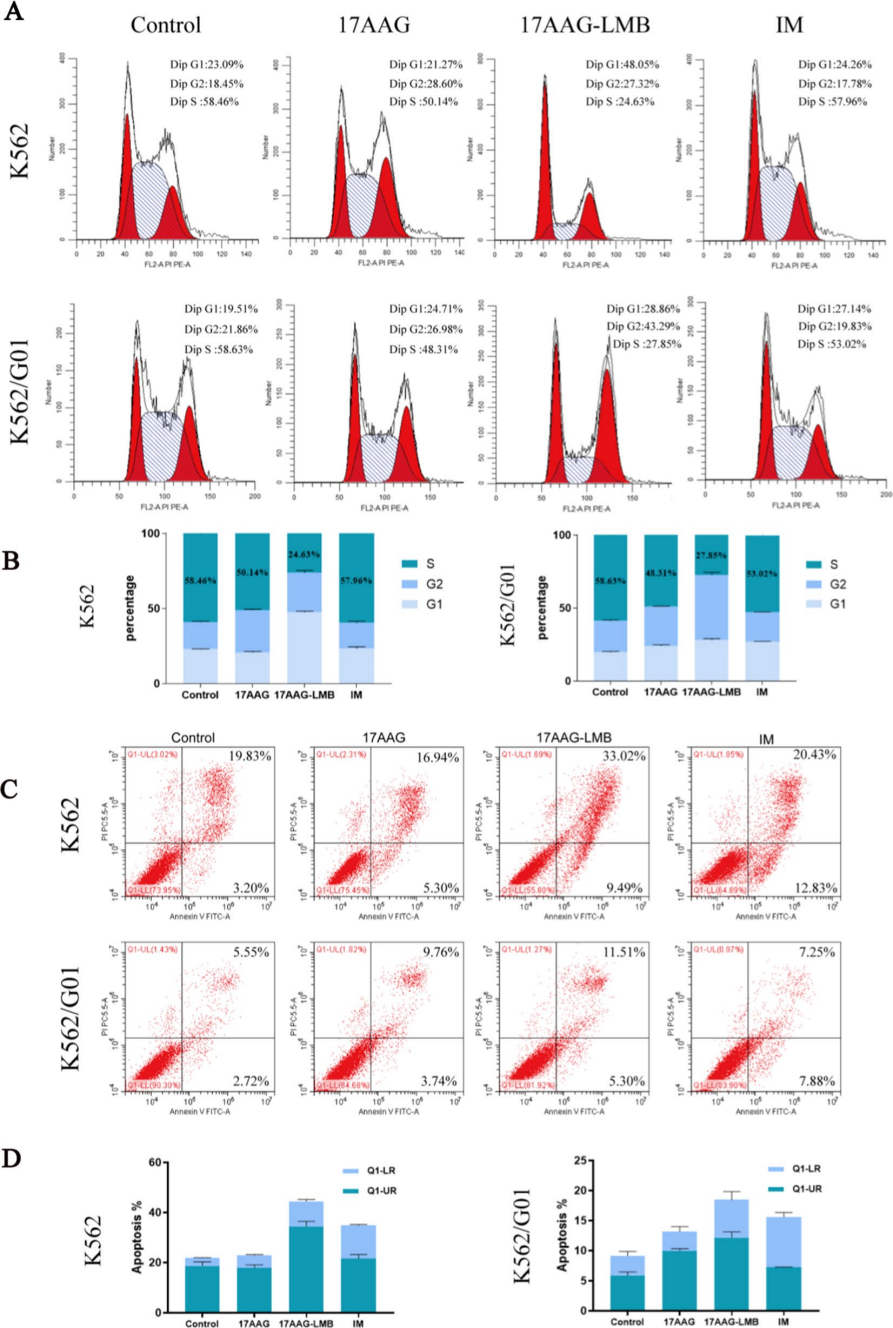
HSP90 inhibitor 17AAG combined with protein nucleus export inhibitor LMB could enhance the killing ability when targeting CML cells. K562 and K562/G01 cells were cultured in control group, 17AAG group, IM group and 17AAG-LMB group respectively **a**, **b** FCM assays the effect of 17AAG combined with LMB inhibitor on CML cell cycle. **c**, **d** FCM assays the effect of 17AAG combined with LMB inhibitor on anti-apoptotic ability of CML cells

Previous data showed that HSP90 inhibitors can induce Bcr-Abl transportation into the nucleus and promote the apoptosis of CML cells, and the inhibitor of protein nuclear export LMB can prevent the transport of Bcr-Abl out of the nucleus. Therefore, it was speculated that the killing ability could be enhanced with the two inhibitors combined targeting CML cells. The results of the proliferation assay showed that 17AAG could significantly inhibit the malignant proliferation of CML cells, and the CML cell apoptosis was more highly promoted in the drug combination group (Fig. 5a, b). The result of colony formation was the same as the proliferation assay. Compared with the group of 17AAG or IM treatment, 17AAG with LMB treatment significantly reduced the proliferation of K562 and K562/G01 cells (Fig. 5c, d). Next, we used flow cytometry analyzer to detect cell cycle and apoptosis, in order to further confirm that HSP90 inhibitor 17AAG combined with protein nucleocapsid inhibitor LMB can enhance the killing ability when targeting CML cells. Cell cycle results showed that compared with other groups, more cells in the drug combination group were blocked in G1-2 phase before DNA replication, with relatively fewer cells in S phase, suggesting that the malignant proliferation capacity of CML cells was significantly reduced with the drug combination treatment (Fig. 6a, b). Apoptosis data showed that a larger proportion of CML cells in the combined group have been induced to apoptosis, and the two drugs combined could enhance the ability of promoting apoptosis of CML cells (Fig. 6c, d).

We have observed the effect of drug combination treatment on the activation of Bcr-Abl and downstream signaling molecules in K562 and K562/G01 cells. Compared with the 17AAG inhibitor and IM treatment alone, the drug combination treatment significantly down-regulated the expression of Bcr-Abl and downstream signaling molecules p-STAT5 and p-AKT (Fig. 5e). The above experiment results indicated that HSP90 inhibitor 17AAG combined with the inhibitor of protein nucleus export LMB could enhance the killing ability when targeting CML cells. And the expression of Bcr-Abl and the downstream signaling molecules are more significantly down-regulated when 17AAG was used in combination with the LMB.

## Discussion

The first line treatment of chronic myeloid leukemia patients is the TKIs, which are applied in clinical treatment with satisfactory efficacy and minor side effects [35]. Nonetheless, CML cells develop TKIs resistance because of the mutations of Bcr-Abl kinase region and pathogenicity of leukemia stem cells. Furthermore, TKIs have inconspicuous effect on some patients who transitioned from the chronic phase to the acute phase [36]. Therefore, identification of novel drug targets needed for CML treatment.

Many progresses have been made in identifying downstream signaling molecules activated by Bcr-Abl during CML pathogenesis [4, 6, 37]. Yet not much is known about the molecular mechanisms sustaining Bcr-Abl localization in the cytoplasm, especially those critical for the development of CML. The localization of Bcr-Abl is an important determinator the development of CML disease, and the CC domain of Bcr-Abl mainly affects its location in the cytoplasm [19, 20]. Protein interactions in leukemia cells can affect protein subcellular localization, hence we speculated that certain proteins in the cytoplasm may bind to the CC domain to retain Bcr-Abl in the cytoplasm [38]. In this study, we screened the target protein HSP90AB1 which is over-expressed in a large variety of cancer cells and belongs to highly conserved ATP-dependent molecular chaperone. HSP90AB1 interacts with transcription factors, cellular kinases, and various molecules to participate in lots of pathophysiological processes of cells [39]. However, the role of HSP90AB1 in the pathogenesis of leukemia is rarely reported. In this report, we have found that HSP90AB1 interacts with the CC domain of Bcr-Abl in the cytoplasm to prevent its degradation. Finding the specific binding site can help us target the Bcr/Abl-HSP90AB1 complex for dissociation. So, we modeled a three-dimensional structure diagram of the Bcr/Abl-HSP90AB1 complex. The direct interaction between the CC domain and the NTD of HSP90AB1 was determined by co-immunoprecipitation. At present, the development and application of HSP90 inhibitors have become a hotspot in tumor therapy, and the targets of inhibitors are also various [25]. The exploration of specific site can make the HSP90 inhibitor more accurate in the selection of therapeutic targets for CML and other Bcr-Abl-addicted disease.

**Fig. 7:**
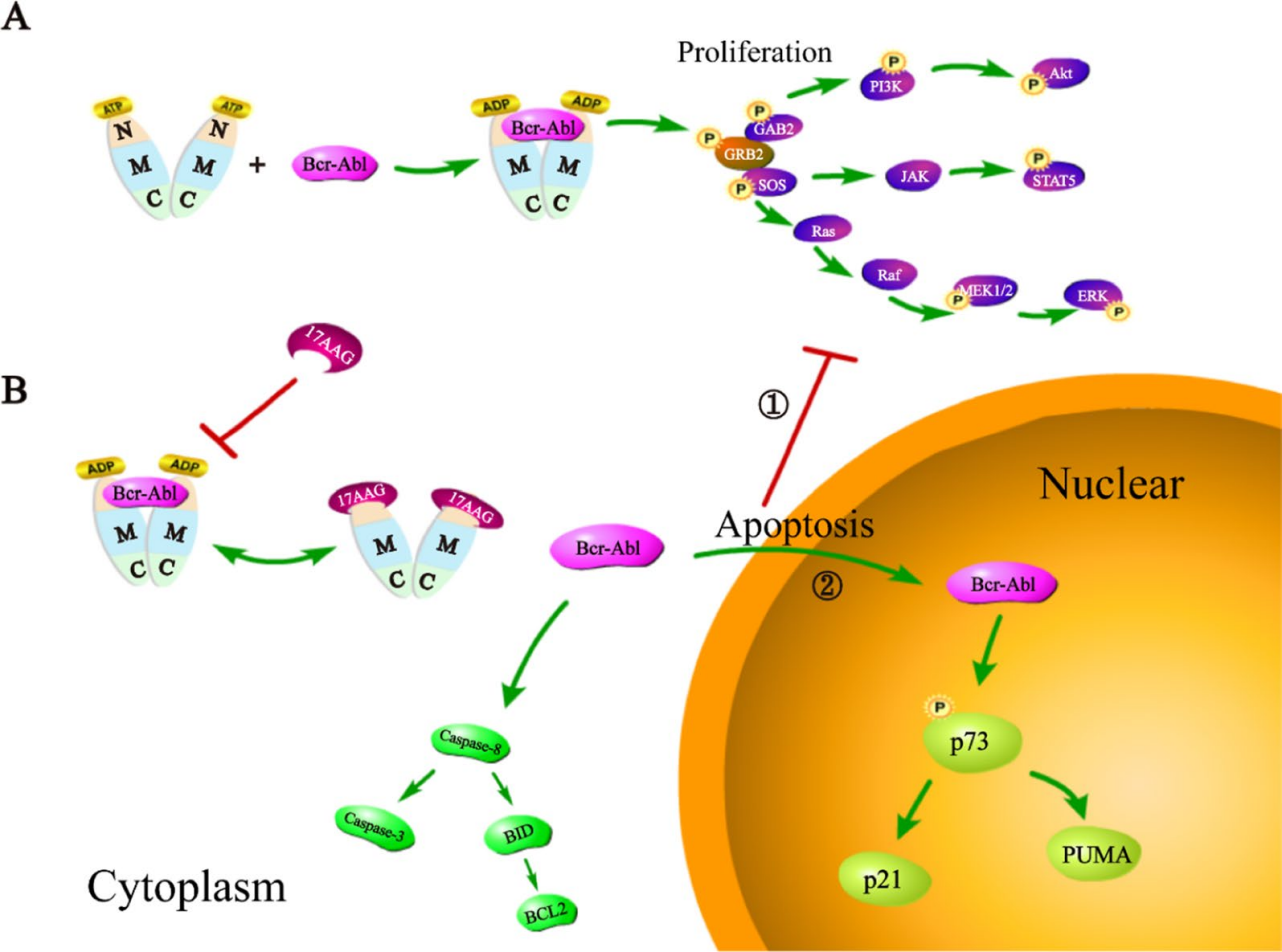
Work model of transporting Bcr-Abl to the nucleus: **a** Normally, Bcr-Abl locates in the cytoplasm and its high tyrosine kinase activity activates multiple downstream signaling pathways, such as JAK/STAT, PI3K/AKT, and RAS/MAPK; **b** When Bcr-Abl is transported into the nucleus under the influence of 17AAG, the specificity binding between 17AAG and HSP90AB1 inhibited the cytoplasmic malignant proliferation signaling though competitively blocking the formation of Bcr/Abl-HSP90AB1 complex; the nucleus-entrapped Bcr-Abl activated p73 and induced the apoptosis of CML cells

The specific localization of Bcr-Abl in the cytoplasm can cause malignant transformation of blood cells, whereas Bcr-Abl induces the apoptosis of CML cells after transporting into the nucleus [15]. In this study, we found the decisive cause for the retention of Bcr-Abl in cytoplasm is the formation of Bcr/Abl-HSP90AB1 complex. For the previously identified binding site, we selected the 17AAG inhibitor that can promote HSP90AB1 dissociation with chaperone protein by antagonizing the NTD ATP function [40]. Interestingly, the Bcr-Abl is transported into the nucleus from the cytoplasm when dissociated with HSP90AB1 under the treatment of 17AAG. The nuclear Bcr-Abl down-regulates the cytoplasmic proliferation signaling activated by the tyrosine kinase of cytoplasmic Bcr-Abl. At the same time, the nuclear Bcr-Abl induces p73 and its downstream targets through c-Abl kinase (Fig. 7). The above experiment results illustrate that the Bcr-Abl can be directed into the nucleus, and Bcr-Abl located in nucleus can induce apoptosis and inhibit the proliferation of CML cells by DNA damage and inhibition of the tyrosine kinase activity. Our research in the early stage conducted a series of studies on the localization of Bcr-Abl, which confirmed its significance to CML. The exploration of the mechanism of specific localization of Bcr-Abl lays the foundation for further study on the pathogenic effect of Bcr-Abl in CML. Furthermore, these data describe a previously neglected strategies, promote the apoptosis of CML cells by inducing the transport of Bcr-Abl into the nucleus.

Targeting protein subcellular localization is considered challenging, because the protein may be transported out of the nucleus after being induced into the nucleus [41]. The Bcr-Abl and c-Abl have similar structural sites, both containing three nuclear localization signals and a nuclear output signal, so it can shuttle between the cytoplasm and nucleus, but is mainly located in the cytoplasm. Considering the nuclear shuttle function of Bcr-Abl, we used the protein nuclear export inhibitor Leptomycin B (LMB) [17]. Our immunofluorescence results displayed that using LMB alone does not affect the localization of Bcr-Abl, but can transport Bcr-Abl into the nucleus in combination with 17AAG. Based on the above results, we then clearly indicated that targeting chronic myeloid leukemia cells, the combination of the two inhibitors can enhance the killing ability. Malignant leukemia cells are particularly sensitive to HSP90 inhibition, leading to the steady development of clinical HSP90 inhibitors [42, 43]. Recently, there are more than thirty positive clinical trials involving the use of HSP90 inhibitors [44, 45]. Conceivably, HSP90 inhibitors will be used as potential alternative therapies to benefit CML patients with Imatinib resistance. However, most current studies on HSP90 inhibitors for the treatment of leukemia have focused on the functional structure of HSP90 and its effect on the phosphorylation or tyrosine kinase activity of the chaperone protein [25, 29, 46]. Following the previous results, our study focuses on the effects of HSP90AB1 on the localization and function of Bcr-Abl, and our findings provide an innovative strategy to develop new therapy of CML. The identification of interaction site in this study can help the development of new HSP90 inhibitors to find the effectively targets [46].

## Conclusions

In summary, we have confirmed that HSP90AB1 plays a critical role in the subcellular localization of Bcr-Abl, which directly affects the development and progression of CML. Based on it, we used both 17AAG and LMB inhibitors to induce Bcr-Abl to be transported into the nucleus. Our data suggest that the combined action of the two inhibitors can enhance the killing ability when targeting CML cells. By finding the important interaction sites between Bcr-Abl and HSP90AB1, our study provides the basis for clinical development of HSP90 inhibitors in treating CML and other Bcr-Abl-addicted malignancies.

## Supplementary Information

**Additional file 1**. **Figure S1.** Effects of CC domain on subcellular localization of BCR-ABL protein. **Table S2.** The primers of HSP90AB1 and HSP90AB1-ΔNTD amplification.

**Additional file 2**. **Table S1.** List of related proteins specifically bound to the CC domain of BCR-ABL protein was screened by mass spectrometry.

## Data Availability

The datasets supporting the conclusions of this article are included within the article and its additional files.

## Abbreviations

Ad: Adenovirus
BCR: Break cluster region
BSA: Bovine serum albumin
CML: Chronic myeloid leukemia
CC domain: The coiled-coil domain
Co-IP: Co-immunoprecipitation
CCK-8: Cell Counting Kit-8
HSP90AB1: Heat shock protein 90 kDa alpha, class B member 1
IM: Imatinib
LMB: Leptomycin B
NTD: N-terminal domain
NLS: Nuclear localization signal
NES: Nuclear export signal
PCR: Polymerase chain reaction
TKI: Tyrosine kinase inhibitor
17AAG: 17-Allylamino-17-demethoxygeldanamycinin
