# Supplementary material for: Effect of HSP90AB1 and CC domain interaction on Bcr-Abl protein cytoplasm localization and function in chronic myeloid leukemia cells
Source: Cell Commun Signal. 2021 Jul 3;19:71. doi: 10.1186/s12964-021-00752-9 (PMC8254927; doi:10.1186/s12964-021-00752-9)

## Effect of HSP90AB1 and CC domain interaction on Bcr-Abl protein cytoplasm localization and function in chronic myeloid leukemia cells

SUPPLEMENTARY MATERIAL


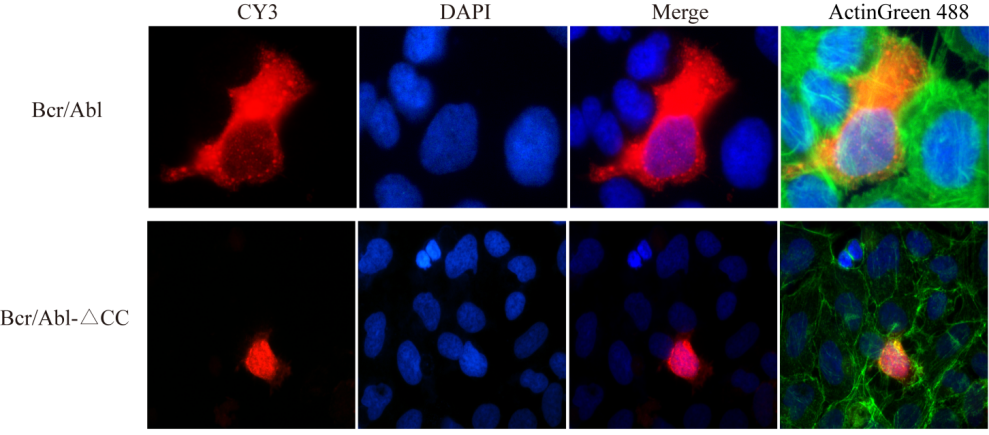


**Supplementary Figure 1:** **Effects of CC domain on subcellular localization of BCR-ABL protein.** The changes of BCR-ABL subcellular localization in 293T cells transfected with pAdTrack-BCR/ABL and pAdTrack-BCR/ABL-ΔCC were observed by indirect immunofluorescence.

**Supplementary Table 1: List of related proteins specifically bound to the CC domain of BCR/ABL protein was screened by mass spectrometry.**

**[Supplementary Table 1-ProteinSummary.xlsx](Supplementary Table 2-ProteinSummary.xlsx)**

**Supplementary Table 2: The primers of HSP90AB1 and HSP90AB1-ΔNTD amplification.**


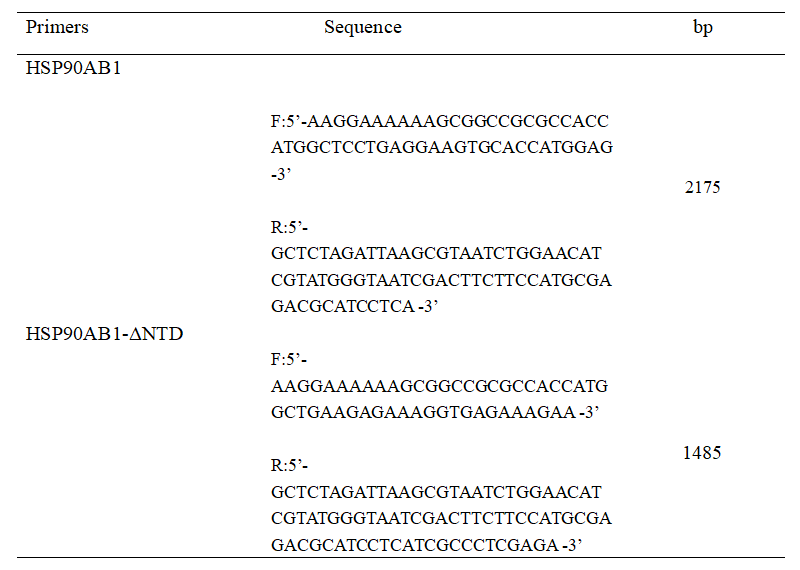

Supplement: Supplementary file 2 — Additional file 1. Figure S1. Effects of CC domain on subcellular localization of BCR-ABL protein. Table S2. The primers of HSP90AB1 and HSP90AB1-ΔNTD amplification. [file 12964_2021_752_MOESM2_ESM.docx]
